# Efficacy and safety of delayed-release dimethyl fumarate in patients newly diagnosed with relapsing–remitting multiple sclerosis (RRMS)

**DOI:** 10.1177/1352458514537013

**Published:** 2015-01

**Authors:** Ralf Gold, Gavin Giovannoni, J Theodore Phillips, Robert J Fox, Annie Zhang, Leslie Meltzer, Nuwan C Kurukulasuriya

**Affiliations:** St. Josef Hospital, Ruhr University, Germany; Queen Mary University of London, Blizard Institute, Barts and the London School of Medicine and Dentistry, London, UK; Multiple Sclerosis Program, Baylor Institute for Immunology Research, Dallas, TX, USA; Mellen Center for Multiple Sclerosis Treatment and Research, Cleveland Clinic, Cleveland, OH, USA; Biogen Idec, Inc., Cambridge, MA, USA; Biogen Idec, Inc., Cambridge, MA, USA; Biogen Idec, Inc., Cambridge, MA, USA

**Keywords:** Delayed-release dimethyl fumarate, multiple sclerosis, newly diagnosed, efficacy, safety

## Abstract

**Background:**

Delayed-release dimethyl fumarate (DMF) demonstrated efficacy and safety in the Phase 3 DEFINE and CONFIRM trials.

**Objective:**

To evaluate delayed-release DMF in newly diagnosed relapsing–remitting multiple sclerosis (RRMS) patients, in a post-hoc analysis of integrated data from DEFINE and CONFIRM.

**Methods:**

Patients included in the analysis were diagnosed with RRMS within 1 year prior to study entry and naive to MS disease-modifying therapy.

**Results:**

The newly diagnosed population comprised 678 patients treated with placebo (*n* = 223) or delayed-release DMF 240 mg BID (*n* = 221) or TID (*n* = 234). At 2 years, delayed-release DMF BID and TID reduced the annualized relapse rate by 56% and 60% (both *p* < 0.0001), risk of relapse by 54% and 57% (both *p* < 0.0001), and risk of 12-week confirmed disability progression by 71% (*p* < 0.0001) and 47% (*p* = 0.0085) versus placebo. In a subset of patients (MRI cohort), delayed-release DMF BID and TID reduced the mean number of new or enlarging T2-hyperintense lesions by 80% and 81%, gadolinium-enhancing lesion activity by 92% and 92%, and mean number of new non-enhancing T1-hypointense lesions by 68% and 70% (all *p* < 0.0001 versus placebo). Flushing and gastrointestinal events were associated with delayed-release DMF.

**Conclusion:**

Delayed-release DMF improved clinical and neuroradiological outcomes relative to placebo in newly diagnosed RRMS patients.

## Introduction

The pathological course of multiple sclerosis (MS) is believed to evolve over time. In the early stages of the disease, autoreactive lymphocytes gain access to the central nervous system, initiating a cascade of events leading to demyelination, axonal transection, and neurodegeneration.^1,2^ In later stages, infiltrative inflammation plays a less prominent role, but extensive neuronal loss and gliosis are evident.^1^ Hence, initiation of MS treatment early in the disease course, when the potential for slowing the accumulation of damage is greatest, could be a clinically meaningful approach. Consistent with this, previous studies with interferon beta and glatiramer acetate (GA) demonstrated an association between early treatment and improved outcomes, such as a prolonged time to conversion from clinically isolated syndrome (CIS) to clinically definite MS (CDMS) and a reduction in the number and volume of lesions on MRI.^3–9^

Delayed-release dimethyl fumarate (DMF) is a novel, oral MS therapeutic studied in people with relapsing–remitting MS (RRMS). In a pre-specified integrated analysis of the Phase 3 DEFINE and CONFIRM trials,^10,11^ delayed-release DMF 240 mg twice (BID) and three times daily (TID) resulted in significant reductions in clinical and magnetic resonance imaging (MRI) activity and demonstrated an acceptable safety profile in RRMS patients over 2 years.^12^ These effects were generally consistent across subgroups of patients stratified by baseline demographic and disease characteristics.^13^

The mechanism by which delayed-release DMF exerts its therapeutic effect in MS is unknown. However, clinical benefits are believed to be related, in part, to activation of the nuclear factor (erythroid-derived 2)-like 2 (Nrf2) pathway^14,15^ and modulation of the expression of pro- and anti-inflammatory cytokines^16–19^ and phase 2 detoxification enzymes.^15,18^ Inflammation and neurodegenerative processes are prominent early in the course of MS, so agents with putative dual anti-inflammatory and neuroprotective effects, such as delayed-release DMF, may be particularly useful.

To examine the efficacy of delayed-release DMF in newly diagnosed patients, a post-hoc analysis of integrated data from DEFINE and CONFIRM was conducted. The newly diagnosed population included patients who had been diagnosed with RRMS within 1 year prior to study entry and were naïve to MS disease-modifying therapy. The analysis included clinical and neuroradiological efficacy endpoints as well as basic safety data (adverse events).

## Methods

### Patients and study design

The designs of the DEFINE and CONFIRM trials have been described in detail elsewhere.^10,11^ Briefly, eligible adult patients (18–55 years) had a diagnosis of RRMS per McDonald diagnostic criteria^20^ and an Expanded Disability Status Scale (EDSS) score of 0–5.0, inclusive.^21^ Further, patients had experienced at least one clinically documented relapse within one year prior to randomization, with a prior brain MRI demonstrating lesion(s) consistent with MS, or at least one Gd+ lesion on a brain MRI scan obtained within 6 weeks prior to randomization.

The primary endpoint of DEFINE was the proportion of patients relapsed at 2 years. The primary endpoint of CONFIRM was the annualized relapse rate (ARR) at 2 years. Additional endpoints included the time to 12-week sustained disability progression, number of Gd+ lesions, number of new or enlarging T2-hyperintense lesions, and number of new T1-hypointense lesions, all at 2 years.

The integrated analysis of DEFINE and CONFIRM was pre-specified prior to the unblinding of CONFIRM and was to be conducted only if the patient populations and treatment effects were similar between the studies. The integrated analysis was considered valid due to the many similarities between DEFINE and CONFIRM, including inclusion/exclusion criteria, regions from which patients were recruited, overall design, measurement criteria, and observed efficacy. Roughly equal proportions of newly diagnosed patients were drawn from each of the pivotal studies.

The newly diagnosed population was defined as patients who had been diagnosed with RRMS per McDonald diagnostic criteria within 1 year prior to study entry^20^ and who were naïve to MS disease-modifying therapy. The 1-year criterion was defined prior to the analysis being conducted and was chosen because the median time since diagnosis of RRMS in the overall treatment-naïve population was 1 year.

### Statistical analysis

This post-hoc analysis was performed on data from the integrated DEFINE and CONFIRM dataset. The pre-planned integrated analysis was finalized prior to unblinding of CONFIRM and required baseline characteristics and treatment effects to be homogeneous across the studies.

The analysis included data from patients in the intent-to-treat (ITT) population (defined as patients who underwent randomization and received at least one dose of study drug) who were randomized to receive placebo or delayed-release DMF BID or TID. Patients randomized to receive GA were excluded because there was no GA comparator arm in DEFINE, and because CONFIRM was not designed to test the superiority or non-inferiority of delayed-release DMF to GA. In general, the analyses were based on all observed data before patients switched to alternative therapies. MRI endpoints were analyzed using ITT patients in the MRI cohort for whom at least one MRI scan was available for analysis. MRI lesion count data post-early withdrawal or post-alternative MS treatment usage were imputed using a constant rate assumption.

Annualized relapse rate (ARR; total number of relapses divided by patient-years in the study, excluding data obtained after patients switched to alternative MS medications) was analyzed with the use of a negative binomial regression model adjusted for baseline EDSS score (≤2.0 vs. >2.0), baseline age (<40 vs. ≥40), study, region (1 [United States], 2 [Western European countries, Canada, Costa Rica, Australia, New Zealand, Israel, and South Africa], or 3 [Eastern European countries, India, Guatemala, and Mexico]) and number of relapses in the year prior to study entry. Regions were pre-defined based on geography, type of health care system, and access to health care in each country. The proportion of patients relapsed was derived using Kaplan–Meier analysis and analyzed with the use of a Cox proportional hazards model with study as a stratifying factor, and adjusted for baseline age (<40 vs. ≥40), region, baseline EDSS score (≤2.0 vs. >2.0), and number of relapses in the year prior to study entry. Disability as measured by time to 12-week confirmed EDSS progression was analyzed using a Cox proportional hazards model with study as a stratifying factor, and adjusted for the following covariates: baseline EDSS score (as a continuous variable), baseline age (<40 vs. ≥40), and region. The odds of having more Gd+ lesions were analyzed using ordinal logistic regression adjusted for study, region, and baseline number of Gd+ lesions. The mean number of new or enlarging T2-hyperintense lesions was analyzed using negative binomial regression adjusted for study, region, and baseline T2-hyperintense volume. The mean number of new non-enhancing T1-hypointense lesions was analyzed using negative binomial regression adjusted for study, region, and baseline volume of T1-hypointense lesions.

## Results

### Study population

The ITT population for the integrated analysis comprised 2301 patients, of whom 678 met newly diagnosed RRMS criteria (332 from DEFINE and 346 from CONFIRM; *n* = 223, 221, and 234 in the placebo, delayed-release DMF BID, and delayed-release DMF TID groups, respectively). A subset of these patients (*n* = 100, 99, and 109 in the placebo, delayed-release DMF BID, and delayed-release DMF TID groups, respectively) comprised the MRI cohort. Baseline demographic and disease characteristics were similar across treatment groups (Table 1). The mean time since diagnosis (standard deviation [SD]) was 0.5 (0.5) years in all treatment groups. The proportion of patients who had received prior treatment with steroids was 7% in the placebo group, 10% in the delayed-release DMF BID group, and 9% in the delayed-release DMF TID group.

**Table 1.** Baseline demographic and disease characteristics of the newly diagnosed population.^a^

Characteristic^b^	Placebo	DMF* BID	DMF* TID
	(*n* = 223)	(*n* = 221)	(*n* = 234)
Age, years	36.5 (9.4)	35.3 (9.4)	36.6 (9.6)
Female, %	70	73	71
Time since first MS symptoms, years	4.3 (5.3)	4.3 (5.8)	3.8 (4.1)
Median (min, max)	2.0 (0, 31)	2.0 (0, 42)	2.0 (0, 23)
Time since diagnosis, years	0.5 (0.5)	0.5 (0.5)	0.5 (0.5)
Median (min, max)	1.0 (0, 1.0)	1.0 (0, 1.0)	1.0 (0, 1.0)
Prior treatment with steroids, %	7	10	9
Relapses in prior year	1.4 (0.6)	1.4 (0.6)	1.5 (0.6)
EDSS score	2.2 (1.1)	2.1 (1.1)	2.0 (1.0)
Gd+ lesion volume,^c^ cm^3^	0.2 (0.4)	0.3 (0.9)	0.1 (0.3)
T2 lesion volume,^c^ cm^3^	8.7 (10.9)	8.5 (9.0)	7.7 (10.7)
T1 hypointense lesion volume,^c^ cm^3^	2.0 (3.6)	2.2 (3.3)	1.6 (2.7)

a The newly diagnosed population included patients who were diagnosed with RRMS within 1 year prior to study entry and naïve to MS disease-modifying therapy; ^b^Values are mean (SD) unless otherwise stated; ^c^MRI cohort only.

Abbreviations: BID, twice daily; EDSS, Expanded Disability Status Scale; Gd+, gadolinium-enhancing; RRMS, relapsing-remitting multiple sclerosis; SD, standard deviation; TID, three times daily.

* DMF, delayed-release DMF

**Figure 1.**
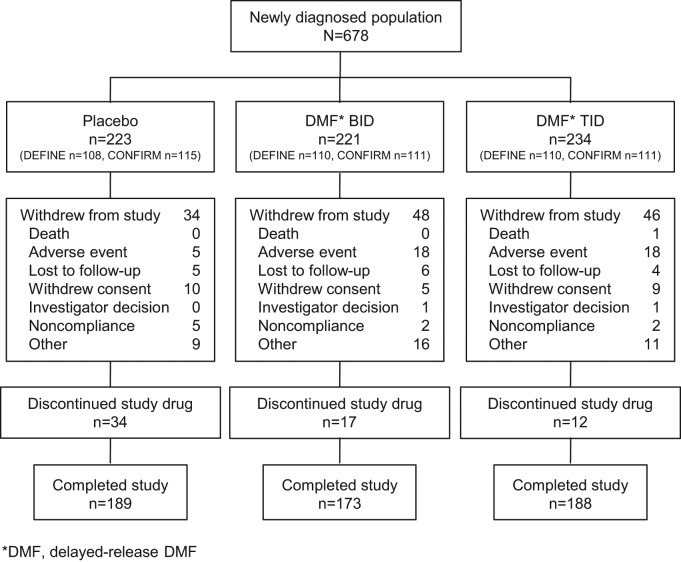
Patient disposition: newly diagnosed population. The ITT population for the integrated analysis comprised 2301 patients, of whom 678 were newly diagnosed (332 from DEFINE and 346 from CONFIRM) and treated with placebo (*n* = 223), delayed-release DMF BID (*n* = 221), or delayed-release DMF TID (*n* = 234). The deaths in the delayed-release DMF TID group were due to a motor vehicle accident and complications of an MS relapse. Abbreviations: BID, twice daily; TID, three times daily.

The proportion of patients who completed the study was 85% in the placebo group, 78% in the delayed-release DMF BID group, and 80% in the delayed-release DMF TID group (Figure 1). The proportion of patients who completed 2 years of study treatment was 70% in the placebo group, 71% in the delayed-release DMF BID group, and 75% in the delayed-release DMF TID group. The mean (SD) number of weeks on study treatment was 80.0 (28.3) in the placebo group, 77.4 (32.6) in the delayed-release DMF BID group, and 79.3 (33.1) in the delayed-release DMF TID group.

### Clinical efficacy

**Figure 2.**
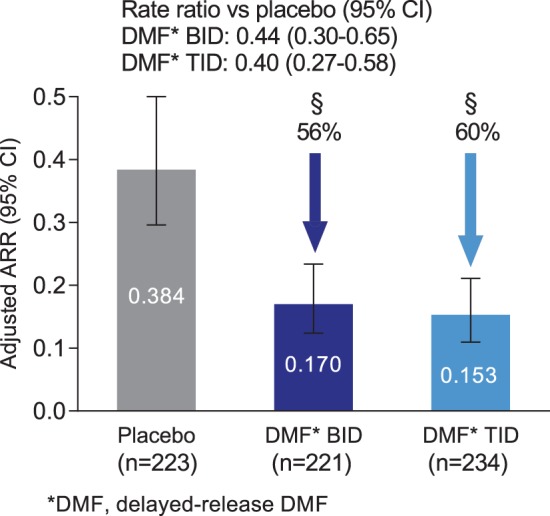
ARR at 2 years in the newly diagnosed population. ARR was calculated using a negative binomial regression model adjusted for baseline EDSS score (≤2.0 vs. *>*2.0), baseline age (*<*40 vs. ≥40), study, region, and number of relapses in the year prior to study entry. The error bars indicate 95% confidence intervals. Abbreviations: ARR, annualized relapse rate; BID, twice daily; CI, confidence interval; TID, three times daily. ^§^*p <* 0.0001 vs. placebo.

**Figure 3.**
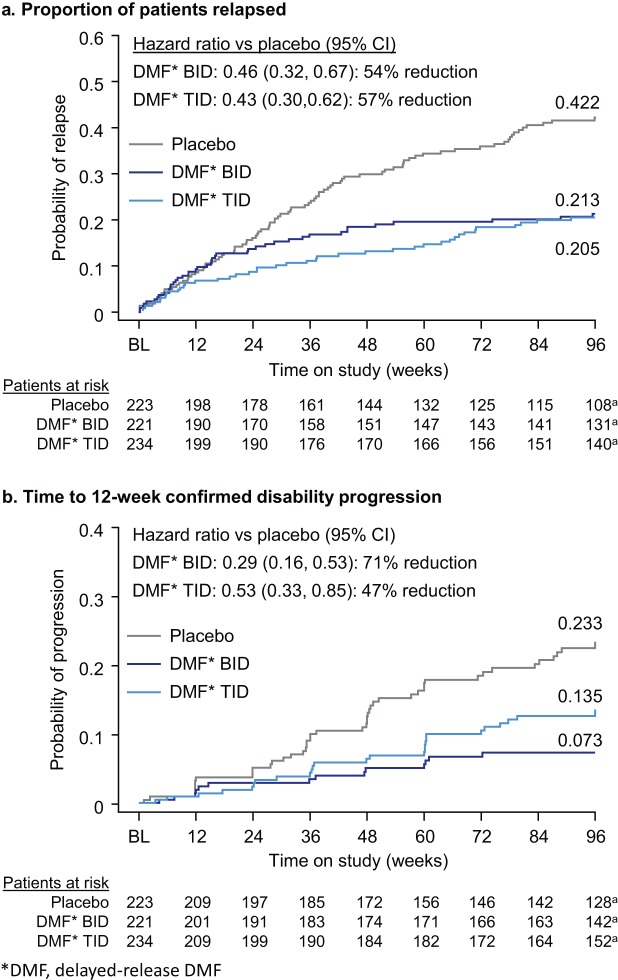
Proportion of patients relapsed and time to 12-week confirmed disability progression at 2 years in the newly diagnosed population. (a) Estimated proportion of patients relapsed at week 96 was derived using Kaplan–Meier analysis. Hazard ratios, 95% CI, and *p* values were based on a stratified Cox proportional hazards model with study as the stratifying variable, adjusted for baseline EDSS score (≤2.0 vs. *>*2.0), baseline age (*<*40 vs. ≥40), region, and number of relapses in the year prior to study entry. (b) Estimated proportion of patients with disability progression at Week 96 was derived using Kaplan–Meier analysis. Hazard ratio, 95% CI, and *p* values were based on a stratified Cox proportional hazards model with study as the stratifying variable, adjusted for baseline EDSS score (as a continuous variable), baseline age (*<*40 vs. ≥40), and region. Abbreviations: BID, twice daily; CI, confidence interval; DR-DMF, delayed-release dimethyl fumarate; TID, three times daily. ^a^Number of patients at risk 5 days prior to the week 96 visit.

The frequency of relapse in the newly diagnosed population was reduced significantly by delayed-release DMF treatment. The ARR at 2 years was 0.38 in the placebo group, 0.17 in the delayed-release DMF BID group, and 0.15 in the delayed-release DMF TID group, representing relative reductions of 56% (BID) and 60% (TID; both *p* < 0.0001 vs. placebo; Figure 2). The risk of relapse was also reduced by delayed-release DMF compared with placebo. On the basis of Kaplan–Meier estimates, the proportion of patients relapsed at 2 years was 0.42 in the placebo group, 0.21 in the delayed-release DMF BID group, and 0.21 in the delayed-release DMF TID group, representing relative reductions of 54% (BID) and 57% (TID; both *p* < 0.0001 vs. placebo; Figure 3(a)).

The risk of 12-week sustained disability progression over 2 years was reduced significantly among newly diagnosed patients receiving delayed-release DMF compared with placebo. On the basis of Kaplan–Meier estimates, the proportion of patients with confirmed 12-week disability progression at 2 years was 0.23 in the placebo group, 0.07 in the delayed-release DMF BID group, and 0.14 in the delayed-release DMF TID group, representing relative reductions of 71% (BID; *p* < 0.0001 vs. placebo) and 47% (TID; *p* = 0.0085 vs. placebo; Figure 3(b)).

### Neuroradiological efficacy

**Figure 4.**
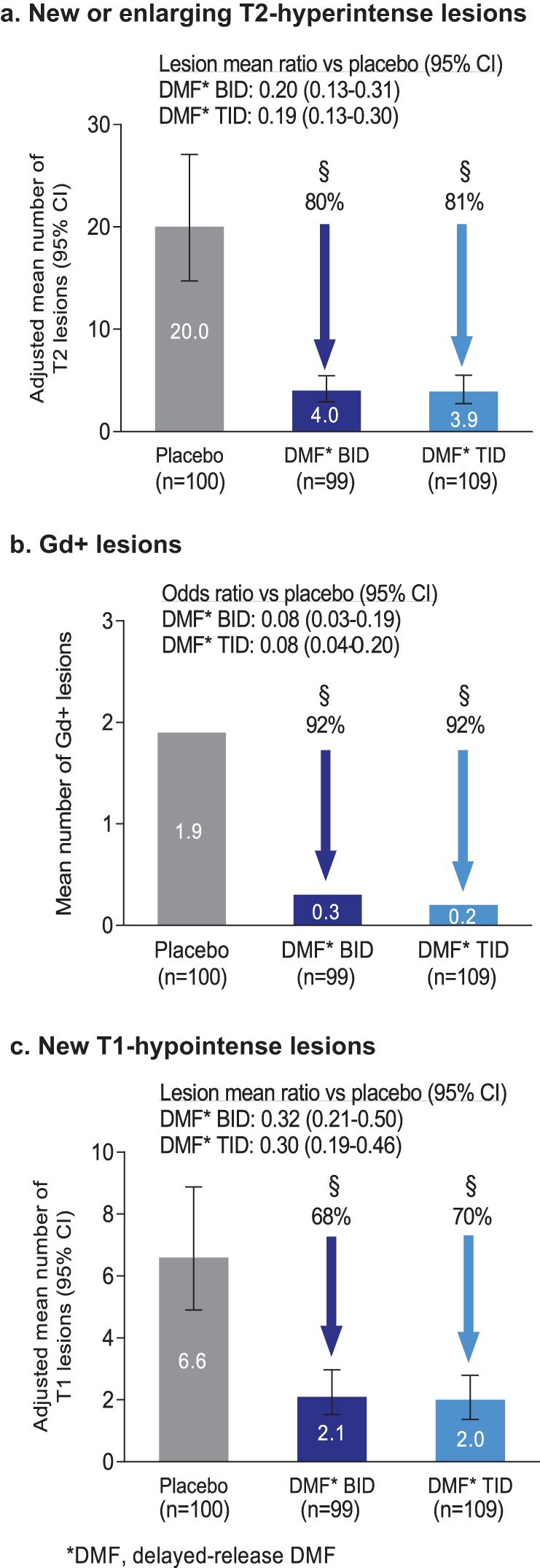
MRI endpoints at 2 years in the newly diagnosed population. (a) The number of new or newly enlarging T2-hyperintense lesions was analyzed using negative binomial regression adjusted for study, region, and baseline volume of T2-hyperintense lesions. Error bars indicate 95% CI. (b) The odds of having more Gd+ lesions were analyzed using ordinal logistic regression adjusted for study, region, and baseline number of Gd+ lesions. Percentages are the reduction in odds of having more Gd+ lesion activity, compared with placebo. (c) The number of new non-enhancing T1-hypointense lesions was analyzed using negative binomial regression adjusted for study, region, and baseline volume of T1-hypointense lesions. Error bars indicate 95% CI. Abbreviations: BID, twice daily; CI, confidence interval; Gd+, gadolinium-enhancing; TID, three times daily. ^§^*p <* 0.0001 vs. placebo

The mean number of new or enlarging T2-hyperintense lesions, odds of having more Gd+ lesions, and mean number of new non-enhancing T1-hypointense lesions were reduced significantly in the newly diagnosed population by delayed-release DMF treatment at 2 years. The adjusted mean number of new or enlarging T2-hyperintense lesions at 2 years was 20.0 in the placebo group, 4.0 in the delayed-release DMF BID group, and 3.9 in the delayed-release DMF TID group, representing relative reductions of 80% (BID) and 81% (TID; both *p* < 0.0001 vs. placebo; Figure 4(a)). The odds of having more Gd+ lesions at 2 years was reduced by 92% in both the delayed-release DMF BID and the delayed-release DMF TID group (both *p* < 0.0001 vs. placebo; Figure 4(b)). The adjusted mean number of new non-enhancing T1-hyperintense lesions at 2 years was 6.6 in the placebo group, 2.1 in the delayed-release DMF BID group, and 2.0 in the delayed-release DMF TID group, representing relative reductions of 68% (BID) and 70% (TID; both *p* < 0.0001 vs. placebo; Figure 4(c)).

### Adverse events

The overall incidence of adverse events in the newly diagnosed population was similar across the placebo (92%), delayed-release DMF BID (97%), and delayed-release DMF TID (95%) groups (Table 2). Adverse events reported more frequently in patients receiving delayed-release DMF compared with placebo (experienced by ≥10% of patients in any group, with an incidence ≥3% higher in either delayed-release DMF group vs. placebo) included flushing, nasopharyngitis, headache, diarrhea, nausea, upper abdominal pain, and abdominal pain (Table 2). The overall incidence of adverse events leading to discontinuation of study treatment was 5% in the placebo group, 12% in the delayed-release DMF BID group, and 11% in the delayed-release DMF TID group.

**Table 2.** Incidence of adverse events experienced by 10% or more of newly diagnosed patients in any treatment group.

Event	Placebo	DMF* BID	DMF* TID
	(*n* = 223)	(*n* = 221)	(*n* = 234)
	*number of patients (percent)*		
Any adverse event	205 (92)	214 (97)	221 (95)
Flushing^a^	8 (4)	86 (39)	65 (28)
Nasopharyngitis^a^	41 (18)	54 (24)	63 (27)
MS relapse	98 (44)	54 (24)	49 (21)
Headache^a^	33 (15)	44 (20)	41 (18)
Diarrhea^a^	21 (9)	32 (15)	47 (20)
Upper respiratory tract infection	28 (13)	31 (14)	29 (12)
Nausea^a^	15 (7)	32 (15)	26 (11)
Urinary tract infection	28 (13)	29 (13)	25 (11)
Proteinuria	22 (10)	23 (10)	29 (12)
Back pain	24 (11)	19 (9)	30 (13)
Upper abdominal pain^a^	11 (5)	20 (9)	28 (12)
Fatigue	21 (9)	20 (9)	26 (11)
Abdominal pain^a^	11 (5)	28 (13)	17 (7)
Arthralgia	21 (9)	22 (10)	23 (10)
Paresthesia	30 (14)	18 (8)	18 (8)
Depression	23 (10)	16 (7)	13 (6)
Pain in extremity	22 (10)	13 (6)	14 (6)

a These events had an incidence ≥3% higher in either delayed-release DMF group vs. placebo.

Abbreviations: BID, twice daily; MS, multiple sclerosis; TID, three times daily.

* DMF, delayed-release DMF

## Discussion

In this post-hoc analysis of integrated data from DEFINE and CONFIRM, delayed-release DMF demonstrated strong efficacy across a broad range of clinical and neuroradiological outcome measures in patients newly diagnosed with RRMS. Over 2 years, both dosing regimens of delayed-release DMF (240 mg BID and TID) significantly reduced the ARR, risk of relapse, proportion of patients with 12-week confirmed disability progression, odds of having more Gd+ lesions, mean number of new or enlarging T2-hyperintense lesions, and mean number of new non-enhancing T1-hypointense lesions, compared with placebo. The effects of delayed-release DMF in the newly diagnosed population were numerically stronger than those seen in the overall ITT population of DEFINE and CONFIRM,^10,11,13^ consistent with findings from previous studies that early intervention with interferons or GA is associated with improved outcomes.^3–9^

From a neuropathological and clinical perspective, the rationale for early intervention is strong. As neurodegenerative effects including axonal transection are observed from the early stages of the disease^2^ and greater frequency of relapse and higher lesion load in early MS are associated with poorer long-term outcomes^22–25^, immediate intervention in newly diagnosed patients may slow the accumulation of damage and progression of disability. Indeed, associations between MS disease activity and long-term clinical prognosis seem to become weaker over time, suggesting an early window of maximal therapeutic opportunity.^23–25^

There is no accepted universal criterion for newly diagnosed or “early” RRMS. Among the criteria that have been used previously are time from symptom onset, time from diagnosis, EDSS score, clinical presentation consistent with CIS, conversion from CIS to CDMS, or a combination of these. In those studies that have used time from diagnosis, the critical interval has varied widely, from immediately following diagnosis^26^ to within 6 months,^27,28^ 2–5 years,^29,30^ or even 8–10 years of diagnosis.^31,32^ As we sought to conduct a robust analysis of a newly diagnosed population, we selected 1 year from diagnosis as our criterion to ensure our study was adequately powered. One year was the median time since diagnosis for the treatment-naïve population of DEFINE and CONFIRM, and it fell clearly within the range of criteria used to characterize the newly diagnosed population in the literature. It should be noted, however, that the patient subgroup evaluated in this report represents patients with early RRMS and not patients with CIS at disease onset.

The recommended dosing regimen of delayed-release DMF is 240 mg BID. The data for 240 mg TID were included here to explore the general consistency of the effects between the doses. In the newly diagnosed population, similar to the ITT population, the effect sizes with delayed-release DMF BID and TID were broadly similar. However, both BID and TID showed a treatment effect on reducing disability progression in the same direction. Although the effect size was different between BID and TID (71% vs. 47% reduction), the confidence intervals of the point estimates overlapped. Therefore, the different effect size was mainly due to data variations of the newly diagnosed population.

The safety and tolerability profile of delayed-release DMF in newly diagnosed patients presented here, while limited in scope, is acceptable and comparable to that seen in the overall integrated safety population of DEFINE and CONFIRM. For example, the overall incidence of adverse events in the newly diagnosed subgroup was 92%, 97%, and 95% in the placebo, delayed-release DMF BID, and delayed-release DMF TID groups, respectively, compared with 93%, 95%, and 94% in the overall safety population.^12^ Flushing, nasopharyngitis, and gastrointestinal events including diarrhea, nausea, and abdominal pain were among the most common adverse events reported by patients treated with delayed-release DMF in both populations.^12^ The incidence of adverse events leading to discontinuation of study treatment in the newly diagnosed subgroup was 5%, 12%, and 11% in the placebo, delayed-release DMF BID, and delayed-release DMF TID groups, respectively, compared with 12%, 14%, and 14% in the overall safety population.^12^

Finally, it should be borne in mind that this is a post-hoc analysis, and, as such, the results should be interpreted cautiously. The study was not powered a priori to analyze the endpoints presented herein in this subgroup of newly diagnosed patients. Therefore, further prospective confirmation is necessary to support our findings.

## Conclusion

This integrated post-hoc analysis suggests strong treatment efficacy of delayed-release DMF in patients with newly diagnosed RRMS, and further supports the use of delayed-release DMF as an oral treatment option in a broad spectrum of people with relapsing MS.
